# Deciphering the Enigmatic Influence: Non-Coding RNAs Orchestrating Wnt/β-Catenin Signaling Pathway in Tumor Progression

**DOI:** 10.3390/ijms241813909

**Published:** 2023-09-10

**Authors:** Xinbing Yang, Yajing Du, Lulu Luo, Xinru Xu, Shizheng Xiong, Xueni Yang, Li Guo, Tingming Liang

**Affiliations:** 1 Jiangsu Key Laboratory for Molecular and Medical Biotechnology, School of Life Science, Nanjing Normal University, Nanjing 210023, China; 221202124@njnu.edu.cn (X.Y.); 221202100@njnu.edu.cn (Y.D.); 211202099@njnu.edu.cn (L.L.); 211202068@njnu.edu.cn (X.X.); 2 Department of Bioinformatics, Smart Health Big Data Analysis and Location Services Engineering Lab of Jiangsu Province, School of Geographic and Biologic Information, Nanjing University of Posts and Telecommunications, Nanjing 210023, China; 1022173308@njupt.edu.cn (S.X.); 1022173309@njupt.edu.cn (X.Y.)

**Keywords:** Wnt/β-catenin, ncRNAs, cancer, drug resistance

## Abstract

Dysregulated expression of specific non-coding RNAs (ncRNAs) has been strongly linked to tumorigenesis, cancer progression, and therapeutic resistance. These ncRNAs can act as either oncogenes or tumor suppressors, thereby serving as valuable diagnostic and prognostic markers. Numerous studies have implicated the participation of ncRNAs in the regulation of diverse signaling pathways, including the pivotal Wnt/β-catenin signaling pathway that is widely acknowledged for its pivotal role in embryogenesis, cellular proliferation, and tumor biology control. Recent emerging evidence has shed light on the capacity of ncRNAs to interact with key components of the Wnt/β-catenin signaling pathway, thereby modulating the expression of Wnt target genes in cancer cells. Notably, the activity of this pathway can reciprocally influence the expression levels of ncRNAs. However, comprehensive analysis investigating the specific ncRNAs associated with the Wnt/β-catenin signaling pathway and their intricate interactions in cancer remains elusive. Based on these noteworthy findings, this review aims to unravel the intricate associations between ncRNAs and the Wnt/β-catenin signaling pathway during cancer initiation, progression, and their potential implications for therapeutic interventions. Additionally, we provide a comprehensive overview of the characteristics of ncRNAs and the Wnt/β-catenin signaling pathway, accompanied by a thorough discussion of their functional roles in tumor biology. Targeting ncRNAs and molecules associated with the Wnt/β-catenin signaling pathway may emerge as a promising and effective therapeutic strategy in future cancer treatments.

## 1. Background

Cancer is a significant global public health issue, with a continuously increasing incidence and mortality rate. It imposes a tremendous burden on both individuals and societies worldwide [1]. The development of cancer is a complex process involving genetic and epigenetic alterations [2,3,4]. Changes in the regulation of certain molecules in cancer-generating genes and signaling pathways may be important information for cancer diagnosis and treatment [5,6]. Moreover, research on this information may further advance the field of cancer diagnostics and targeted therapies. The Wnt/β-catenin signaling pathway predominantly regulates essential biological functions, such as early embryo development, tissue regeneration, cell proliferation, differentiation, migration, and apoptosis [7,8]. It plays a vital role in tumor initiation and treatment. The previous literature has reported that the Wnt/β-catenin pathway is a key driving factor in cancer development and a hot target in current cancer therapeutics [9,10,11,12]. Disruption of this signaling pathway is closely associated with the occurrence and progression of various types of cancer [13,14,15].

Recent studies have shown that non-coding RNAs (ncRNAs), as well as Wnt/β-catenin signal pathways, play an active role in the development and progression of cancer [16]. ncRNAs are a unique class of transcripts that lack protein-coding capacity [17]. Currently identified ncRNAs primarily fall into three categories: microRNAs (miRNAs), long non-coding RNAs (lncRNAs), and circular RNAs (circRNAs) [18,19]. Previously considered as “junk DNA”, ncRNAs have been increasingly recognized for their central role in gene regulation and their significant impact on tumor pathways under physiological and pathological conditions [19,20,21,22]. The activation and silencing of the Wnt/β-catenin signaling pathway, as well as abnormal expression of ncRNAs, are closely linked to cancer development. Many existing experimental studies have demonstrated that aberrant expression of ncRNAs can affect behaviors, such as cancer migration and invasion, by regulating the activation and silencing of this Wnt/β-catenin signaling pathway.

This review aims to summarize the impact of abnormal expression of ncRNAs related to the Wnt/β-catenin signaling pathway on cancer initiation and development. It intends to elucidate the regulatory relationship between ncRNAs and the Wnt/β-catenin pathway. Additionally, it aims to provide an overview of the current application value and potential of ncRNAs in cancer diagnosis and treatment, thereby offering further insights for future research on the role of ncRNAs in tumor initiation and therapeutics. The ncRNAs data were retrieved through a comprehensive search on NCBI (PubMed), employing specific keywords, such as “ncRNA”, “Wnt/β-catenin”, and various types of cancer like “breast”, “lung”, “colorectal”, “prostate”, and “gastric cancer.” The search results were further filtered to encompass publications between the years 2019 and 2023.

## 2. Introduction to the Wnt Signaling Pathway and the Involvement of ncRNAs in Cancer

ncRNAs, a special class of RNA transcripts, do not encode proteins but exhibit high specificity and stability, making them crucial for assessing the occurrence and progression of cancer [23,24]. Based on their functions, ncRNAs are typically categorized into housekeeping RNAs and regulatory RNAs [25]. Regulatory RNAs mainly include miRNAs, lncRNAs, and circRNAs [23,24,25,26]. miRNAs are extensively studied small non-coding RNAs (sncRNAs) in eukaryotes and consist of approximately 22 nucleotides (nt) in length [27]. miRNAs recognize target mRNAs through complementary base pairing, leading to the degradation of the targeted mRNA or the silencing of mRNA translation by the RNA-induced silencing complex (RISC), thereby inhibiting gene expression [27,28]. A single miRNA can regulate multiple different genes, and multiple miRNAs can jointly regulate a single gene. This intricate regulatory network is increasingly associated with the abnormal expression of miRNAs and the progression of various cancers [29,30].

Both lncRNAs and circRNAs are over 200 nt in length; can be transcribed from the exon, the intron, the intergenic region of the gene, and the 5/3-untranslated region; and fold into complex secondary structures that facilitate their interactions with DNA, RNA, and proteins [31,32,33,34,35]. Through various mechanisms, lncRNAs and circRNAs regulate gene expression. They can serve as competitive endogenous RNAs (ceRNAs) that act as decoys or sponges, competitively sequestering miRNAs and forming intricate lncRNA–miRNA–mRNA and circRNA–miRNA–mRNA networks, thereby regulating the expression levels of downstream target genes. Additionally, they can act as scaffolds to modulate protein–protein interactions and downstream signaling pathways. Recent studies have also identified the critical role of lncRNA-mediated regulation of Wnt/β-catenin signaling in epithelial–mesenchymal transition (EMT) processes in human tumors [36]. Over the past decade or so, ncRNAs have changed their role from “junk” transcription products to functional regulatory molecules that mediate cellular processes and participate in various cellular functions in many cancers through various signaling pathways, including Wnt/β-catenin [37]. For example, LINC01133 acts as a ceRNA for miR-106a-3p, regulating APC expression and the Wnt/β-catenin pathway, thereby inhibiting the progression and metastasis of gastric cancer cells [38]. Downregulation of miR-125b and inhibition of Wnt/β-catenin signaling inhibit proliferation and migration in TNBC [39]. Additionally, circ_0082182 activation of the Wnt/β-catenin pathway by absorption of miR-411 and miR-1205 through sponge action promotes malignant progression of colorectal cancer cells [40].

The Wnt signaling pathway is an important pathway widely present in multicellular organisms. It plays a crucial role in biological development, tissue regeneration, and various diseases, particularly in the occurrence and progression of cancer [41,42,43]. Furthermore, the composition of this pathway is highly conserved throughout evolution. The core genes of this pathway include Wnt, frizzled receptors, disheveled (DSH), GSK-3β, AXIN and β-catenin, among others. Among them, the Wnt protein is a critical entity that guides the signal transduction in this pathway.

Upon activation of the Wnt signaling pathway, downstream signaling requires the further activation of DSH. DSH consists of three distinct structural domains: the amino-terminal DIX domain, the central PDZ domain, and the carboxy-terminal DEP domain. These three domains of DSH play a crucial switch-like role in the Wnt signaling pathway [44] and determine the branching of the Wnt signal into different downstream pathways. The branching results in the formation of different Wnt pathways, including the Wnt/β-catenin or classical Wnt signaling pathway, the Wnt/Ca^2+^ signaling pathway, and the planar cell polarity (PCP) pathway [45,46]. Each of the three branches has different functions and participates in the regulation of various physiological activities.

Currently, research on the Wnt pathway primarily focuses on the Wnt/β-catenin signaling pathway branch. This pathway can determine cell fate, cell proliferation, cell survival, and intercellular interactions [47]. Dysregulation of this pathway is associated with the development of various malignancies [15,48,49,50,51,52,53]. For example, upregulation of the Wnt/β-catenin signaling pathway induced by gene mutations or abnormal activation of Wnt receptors can lead to the carcinogenesis of several tissues, such as the liver, lung, pancreas, and colon [54,55,56,57,58,59].

**Figure 1:**
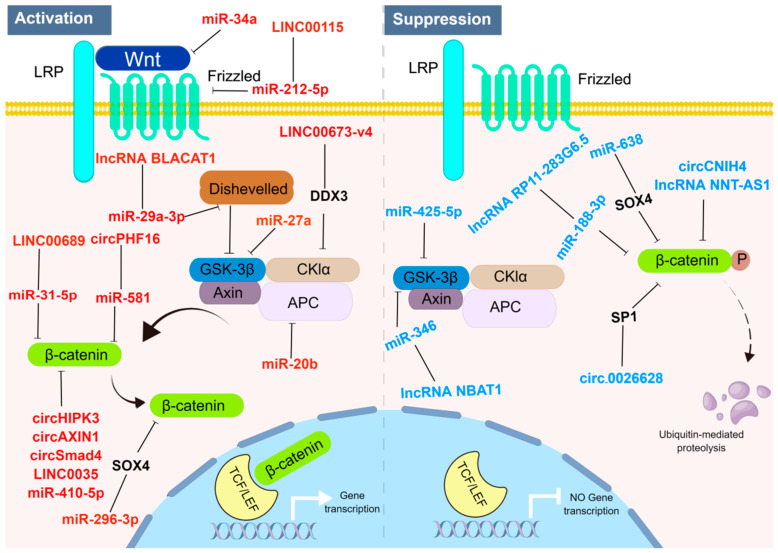
Regulation of Wnt/β-catenin Signaling Pathway by ncRNAs: Insights into Activation and Suppression Mechanisms (By Figdraw). “Activation State”: when Wnt binds to drizzled and LRP receptors, cytoplasmic protein DVL is activated, leading to the inhibition of GSK3β. Subsequently, stabilized β-catenin translocates into the nucleus and interacts with TCF/LEF transcription factors, resulting in target gene transcription. In this activation mechanism, specific ncRNAs, such as miR-34a and miR-27a, play pivotal roles. They interact with the promoter region of Wnt genes, promoting the transcription and expression of Wnt proteins, thus enhancing Wnt protein levels. Additionally, they interact with negative regulators in the Wnt/β-catenin signaling pathway, such as Axin and GSK-3β, reducing their degradation effect on β-catenin. Furthermore, ncRNAs like circHIPK3 also modulate key genes in the pathway, decreasing their degradation effect on β-catenin and promoting the activation of the Wnt/β-catenin signaling pathway, thereby enhancing its activity. “Suppression State”: in the absence of WNT ligands, the destruction complex of β-catenin consists of AXIN, CK1α, GSK3β, and APC, which phosphorylate β-catenin for subsequent ubiquitin–proteasome degradation. In the suppression mechanism, certain ncRNAs, such as miR-425-5p and circ_0026628, play essential roles. They interact with negative regulators in the Wnt/β-catenin signaling pathway, such as Axin and GSK-3β, and enhance their degradation effect on β-catenin. This leads to increased degradation of β-catenin, resulting in the inhibition of signaling pathway activity. Moreover, ncRNAs like circ_0026628 can also interact with regulatory factors associated with downstream genes in the Wnt/β-catenin signaling pathway, inhibiting their transcriptional activity and reducing the transcription levels of downstream genes. These interactions further enhance the degradation of β-catenin, ultimately influencing the activity state of the signaling pathway. ncRNAs highlighted in red font signify the activation of the Wnt signaling pathway, whereas those in blue font represent inhibition of the Wnt signaling pathway.

The interplay between ncRNAs in regulating the Wnt/β-catenin signaling pathway is depicted in Figure 1. Wnt serves as the major regulatory factor for β-catenin. β-catenin is a member of a family consisting of 19 cadherin proteins and can regulate both dependent and independent signaling pathways of β-catenin [60]. The distinct feature of the Wnt/β-catenin signaling pathway is the formation of a complex involving Wnt, its core receptor complex, and members of the frizzled (FZD) protein family, which regulates the degradation and protection of β-catenin during signal transduction [61]. In the presence of Wnt protein ligands, Wnt binds to FZD receptors and co-receptor LRP to form a complex. The LRP receptor is then phosphorylated by CK1α and GSK3β, which recruit DSH at the cell membrane, where they aggregate and activate. Once activated, they inhibit the activity of the degradation complex, allowing unphosphorylated β-catenin to translocate to the nucleus and accumulate. It then binds to LEF-1/TCF4 and other co-regulatory factors in a tissue-specific manner to promote the transcription of target genes. This activation of downstream target genes drives cell-cycle progression or generates abnormal proteins, leading to cellular carcinogenesis [62]. In the absence of stable Wnt ligands, β-catenin in the cytoplasm is recruited to a complex involving adenomatous polyposis coli (APC) and Axin, promoting the phosphorylation of β-catenin by casein kinase 1α (CK1α) and glycogen synthase kinase 3β (GSK3β). Phosphorylation of specific sites leads to the binding of E3 ubiquitin protein ligase subunits, targeting β-catenin for proteasomal degradation, thus maintaining low levels of β-catenin in the cytoplasm [55]. Without nuclear translocation of β-catenin, in the absence of β-catenin, inhibitory complexes containing T-cell factor/lymphoid enhancer factor (TCF/LEF) and transducin-like enhancer protein (TLE/Groucho) recruit histone deacetylase (HDAC) to suppress target gene transcription, leading to the inhibition of downstream target genes [63,64,65,66]. In more than half of cancer cases, such as colorectal cancer, breast cancer, liver cancer, and melanoma, β-catenin accumulates in the nucleus or cytoplasm [67,68,69,70].

An increasing body of research has demonstrated the significant regulatory roles of ncRNAs in the occurrence and progression of various cancers through the modulation of the Wnt signaling pathway. The Wnt pathway is known to play crucial roles in cell differentiation, proliferation, and self-renewal in a variety of cells. Disturbances in the Wnt pathway have been closely associated with the development and progression of several cancers, including liver cancer, colon cancer, breast cancer, and prostate cancer. As a class of RNA molecules that do not encode proteins, ncRNAs have garnered extensive attention regarding their mechanisms and regulatory modes in cancer. The specific mechanisms of ncRNAs vary among different types of cancer. For example, in bladder urothelial carcinoma, miRNA-139-3p inhibits malignant progression by targeting KIF18B and inactivating the Wnt/β-catenin pathway [71]. In contrast, in liver cancer, miR-342 promotes cell proliferation and apoptosis in hepatocellular carcinoma through the Wnt/β-catenin signaling pathway [72]. CircRNAs and lncRNAs have also received widespread attention for their role in cancer development. For instance, the expression levels of the lncRNA HOTAIR are elevated in various cancers. It can promote the proliferation and invasion of renal cell carcinoma by regulating the Wnt/β-catenin pathway and influencing cisplatin resistance [73]. Additionally, other Wnt signaling-pathway-related genes, such as AXIN2, DKK, and SFRP, as well as various classes of ncRNAs that regulate them, are closely associated with the occurrence and progression of multiple cancers. In addition to the well-known factors of the Wnt/β-catenin signaling pathway, certain genes seemingly unrelated to this pathway can also become major targets of ncRNA regulation. ncRNAs can modulate the expression of these genes through various mechanisms such as gene substrate competition, transcriptional regulation, and signal transduction regulation. This ultimately leads to direct or indirect effects on the β-catenin pathway, even if the ncRNAs themselves are not members of the pathway. The recent literature has reported that CREPT [74], as a co-activator, can enhance the transcriptional activity of the β-catenin–TCF4 complex stimulated by Wnt signaling. Additionally, miR-449b-5p has been identified as a regulator of Wnt/β-catenin signaling by targeting CREPT [75]. These findings suggest that the regulatory network of the Wnt/β-catenin pathway is more complex than previously thought. It highlights the importance of considering the involvement of seemingly unrelated genes and ncRNAs in the modulation of this signaling pathway. Further understanding the mechanisms through which ncRNAs regulate non-canonical target genes provides valuable insights into the intricate regulation of the cell and the transmission of Wnt/β-catenin signaling. The identified roles of CREPT and miR-449b-5p in this context open up new avenues for investigating the potential therapeutic implications of targeting ncRNA-mediated regulation in Wnt/β-catenin signaling.

Despite the need for further research to gain a deeper understanding of the mechanisms by which ncRNAs contribute to cancer, the study of ncRNAs and their regulation of the Wnt pathway hold great significance for cancer diagnosis, treatment, and the development of personalized medicine in the future.

## 3. The Roles and Mechanisms of ncRNAs Involved in the Wnt/β-Catenin Signaling Pathway in Tumors

In the past few years, ceRNA has been detected in Wnt/β-Catenin signaling-pathway-related cancers, exhibiting increasingly important regulatory roles (Table 1), particularly in breast cancer, lung cancer, colorectal cancer, prostate cancer, and gastric cancer.

### 3.1. The Impact of the Interplay between ncRNAs and the Wnt/β-Catenin Signaling Pathway on Breast Cancer

Breast cancer (BC), as a common disease threatening women’s health, has been receiving much attention in terms of its treatment and prognosis [1]. The Wnt/β-catenin signaling pathway plays a key regulatory role in the initiation and progression of BC. In recent years, more and more studies have revealed that this pathway is widely regulated by ncRNA. These ncRNAs assume important functions in BC, including regulation of Wnt/β-catenin activation, regulation of the expression of related genes, and adjustment of biological behaviors, such as cell proliferation, invasion, and metastasis.

As a small RNA molecule, miRNA can regulate gene expression at the post-transcriptional level by interacting with mRNA targets. It was found that miR-296-3p downregulated SOX4 by targeting the Wnt/β-catenin signaling pathway to produce anti-tumor effects in TNBC [76], while miR-638 downregulation is associated with poor prognosis in BC patients, and its suppression of HOXA9 and the Wnt/β-catenin signaling pathway can inhibit BC progression [77]. Additionally, lncRNAs can serve as regulators of miRNAs involved in the regulation of the Wnt/β-catenin signaling pathway. For instance, lncRNA MICAL2-1 can bind with miR-25 to suppress the development of BC by regulating DKK3 and inhibiting the activation the Wnt/β-catenin signaling pathway [78]. TMED3, a member of the transmembrane emp24 domain-containing (TMED) protein family, plays a crucial role in protein trafficking and secretion. Modulating TMED3 expression can potentially influence the availability and localization of components involved in the β-catenin pathway. Previous studies have shown that overexpression of TMED3 increases the expression of β-catenin and Axin2, as well as the complex formation of downstream target genes in the Wnt/β-catenin signaling pathway. In a BC study, researchers identified lncRNA RP11-283G6.5 as a modulator of the Wnt/β-catenin pathway. They found that RP11-283G6.5 restricts the progression of BC by regulating the miR-188-3p/TMED3 axis within the Wnt/β-catenin signaling pathway [79], while circ_0008784 can regulate the pathway and promote TNBC cell progression via miR-506-3p-mediated regulation of CTNNB1 [80]. It was also reported that LINC00511-encoding small peptide LINC00511-133 aa restricts apoptosis by regulating the expression levels of Wnt/β-catenin signaling-pathway-related proteins Bax, c-myc, and CyclinD1 and promotes β-catenin into the nucleus, ultimately affecting BC cell invasion and stemness [81].

In addition, ncRNAs also play an important role in the treatment and resistance of BC. Apatinib can inhibit BC development by blocking the Wnt/β-catenin signaling pathway through downregulation of lncRNA ROR [82]. In BC cells surviving after chemotherapy, chemotherapeutic agents, such as doxorubicin or paclitaxel, can activate the EZH2/STAT3 axis in BC cells, leading to the secretion of exosomes rich in miR-378a-3p and miR-378d, which can induce drug resistance by targeting DKK3 and activating the Wnt/β-catenin signaling pathway [83]. Additionally, FSTL1 in BC cells can activate the Wnt/β-catenin signaling pathway via integrin β3, and miR-137 can downregulate FSTL1 mRNA and protein levels to form a miR-137/FSTL1/integrin β3/Wnt/β-catenin signaling axis, regulating BC stemness and chemoresistance [84].

Ultimately, the ncRNAs associated with the Wnt/β-catenin signaling pathway plays an important regulatory role in BC. These ncRNAs affect the proliferation, invasion, metastasis, and stem-cell properties of BC cells by directly or indirectly regulating Wnt/β-catenin signaling. Moreover, they are involved in the regulation of drug resistance and immune escape in BC cells. A deeper understanding of the mechanisms of these ncRNAs in BC will help to reveal the molecular basis of BC development and provide new strategies and targets for precision therapy and personalized medicine. However, many questions still need to be further investigated, including the interaction of ncRNAs with the Wnt/β-catenin signaling pathway, its regulatory effects in the tumor microenvironment, and its potential and limitations in clinical application. Through continuous intensive research, we hope to reveal more details about the occurrence and development of BC and provide more precise methods and strategies for its treatment and prevention. 

### 3.2. The Effect of the Interaction between ncRNA and Wnt/β-Catenin Signaling Pathway on Lung Cancer

The study of Wnt/β-catenin signaling pathway in lung cancer (LC) is increasingly valued, and the role of the ncRNA involved in this tumor is gradually revealed. Lung cancer is a highly heterogeneous and lethal disease, and understanding the function and mechanism of the Wnt/β-catenin signaling-pathway-related ncRNAs in lung cancer is essential to revealing the molecular basis of its occurrence and development and for the development of new therapeutic strategies [85]. 

MiR-590 is a type of miRNA that commonly exhibits abnormal expression in various tumors. Ma et al. found that this miRNA is downregulated in non-small-cell LC (NSCLC) and closely related to patient prognosis [86]. Overexpression of miR-590 can inhibit the proliferation and invasion of NSCLC cells by targeting GAB1 [87]. On the other hand, miR-590 also regulates LC progression by targeting YAP1 and inhibiting the activation of the Wnt/β-catenin signaling pathway [88].

Another ncRNA related to the Wnt/β-catenin signaling pathway is lncRNA SNHG11 [89]. Its expression is highly upregulated in LC and plays a role in promoting LC cell proliferation, migration, invasion, and the epithelial–mesenchymal transformation process. The ncRNAs could promote LC progression via the activation of the Wnt/β-catenin signaling pathway in two different modes while inhibiting apoptosis. Moreover, lncRNA SNHG11 overexpression was correlated with the poor prognosis, TNM stage, and tumor size of LC patients. LncRNA FLVCR1-AS1 is another ncRNA that was found to regulate the Wnt/β-catenin signaling pathway, inhibiting the proliferation, migration, and invasion of LC cells by suppressing the activity of this pathway [90]. When lncRNA FLVCR1-AS1 is silenced, the expression levels of CTNNB1, SOX4, CCND1, CCND2, c-MYC and β-catenin nuclear protein decrease in LC cells.

Several other ncRNAs have also been found to be intimately involved in the initiation and progression with LC [91,92,93,94,95,96,97]. For example, circEIF3I positively regulates NOVA2 expression by sequestering miR-1253 and further modulates the activity of the Wnt/β-catenin signaling pathway, promoting LC progression. In addition, the low expression of miR-448 inhibits the activation of the Wnt/β-catenin signaling pathway, promoting the proliferation of LC cells and platinum resistance. Furthermore, the suppression of miR-140-5p induces the activation of the Wnt/β-catenin signaling pathway, ensuring platinum resistance in LC cells.

Thus, Wnt/β-catenin signaling activation favors lung cancer progression. Potential therapeutic strategies could be focused on targeting Wnt signaling or ncRNA as an upstream mediator. The limitations of the current experiments are their emphasis on oncogenic ncRNA and the increased effort required to identify the tumor-suppressor ncRNAs regulating Wnt signaling in lung cancer.

### 3.3. The Effect of the Interaction between ncRNA and Wnt/β-Catenin Signaling Pathway on Colorectal Cancer

Colorectal cancer (CRC) is a common cancer with a high incidence in both men and women [1]. Despite advances in surgery, radiation, and chemo, there are still many challenges in the treatment of CRC [98]. The Wnt signaling pathway is thought to be the major disruption pathway in this malignancy. The ncRNA is thought to promote CRC pathogenesis by triggering or hindering the Wnt signaling pathway.

MiRNAs are a class of short-chain RNAs with a length of 20–24 nucleotides that have been found to be important regulators in various tumors. Studies have found that miRNA-621 directly targets LEF1 and inhibits Wnt/β-catenin signaling, thereby playing a role in the inhibition of metastasis in CRC [99]. Other than miRNAs, lncRNAs and circRNAs also play important regulatory roles in CRC. LINC00665 upregulates CTNNB1 to activate the Wnt/β-catenin signaling pathway and stimulate the tumorigenicity of CRC, promoting tumor progression in the colon [100], and SNHG 4 promotes the progression of colorectal cancer by enhancing RNF15 mRNA stability and activating the Wnt/β-catenin pathway [101]. Conversely, hsa_circ_0026628 plays an inhibitory role in CRC cell proliferation, migration, and EMT focusing on SP1 to inhibit the Wnt/β-catenin pathway [102]. Furthermore, LNC00689 participates in the proliferation, chemotherapy resistance, and metastasis of CRC through the miR-31-5p/YAP/β-catenin axis [103]. In CRC tissues and cells, the downregulation of LINC00689 and upregulation of its downstream target miR-31-5p degrades LATS2, activates the YAP1/β-catenin signaling pathway, and accelerates the development of CRC. Therefore, LINC00689 may be an ideal potential target and positive prognostic factor for 5-fluorouracil (5-FU) chemotherapy in the treatment of CRC. Similarly, hsa_circ_0001666 interferes with Wnt/β-catenin signaling by targeting PCDH10 through miR-576-5p, inhibiting CRC cell proliferation, invasion, and metastasis; inducing apoptosis; and suppressing EMT and stem cells [104]. These results indicate that lncRNAs and circRNAs participate in the regulation of the Wnt/β-catenin signaling pathway to regulate the biological behavior and chemotherapy sensitivity of CRC.

Overexpression of SNHG15, located on chromosome 7p13, has been associated with low survival rates in many human malignancies, including CRC [105,106]. Recent studies have shown that SNHG15 has a critical role in regulating various pathways associated with tumor progression, including Wnt/β-catenin signaling pathway and EMT regulation of CRC [107]. SNHG15 mediates CRC by regulating target gene multiplication, aggression, and migration, along with resistance to colorectal cancer therapy [108,109,110]. Alternative research has also shown that overexpression with SNHG15 adds to drug resistance in CRC cells through strong binding with the translocator MYC [109]. Additionally, SNHG15 is further associated with EMT and colon cancer proliferation through slug–protein interaction [110]. These results suggest that SNHG15 has an essential function to play in CRC by participating in the regulation of the Wnt/β-catenin signaling pathway and other pathways.

Cancer pathogenesis is correlated with lncRNA expression, and several approaches have been proposed to target lncRNA for cancer therapy [111]. One such approach is the knockdown of lncRNA by the post-transcriptional RNA degradation pathway. Specific methods include targeting lncRNA using small interfering RNA (siRNA) through dichotomous and agonamic acid (AGO)-dependent cleavage pathways. Another method is to use antisense oligonucleotides (ASOs) with chemical modifications or gaps to degrade RNA by forming RNA–RNA or RNA–RNA hybrids by the ribonuclease H (RNase-H) mechanism. Furthermore, transcription blockade is an alternative strategy that could permanently or partially delete lncRNA target regions in lncRNA genomic sites by the CRISPR/Cas 9 editing system or block lncRNA expression by insertion of a polyadenylation signal. Modified ASO or RNA-bound small molecules can also be used to interfere with lncRNA–protein interactions, thus enabling inhibition of lncRNA function. Each of these methods can be applied to SNHG15 in CRC to improve the impact on CRC progression.

### 3.4. The Effect of Interaction between ncRNA and Wnt/β-Catenin Signaling Pathway on Prostate Cancer

Prostate cancer (PCa) is a prevalent malignant tumor and remains one of the primary threats to male health [112]. Although various treatment approaches have emerged in recent years, the prognosis and survival rates for this disease remain suboptimal [113,114]. Several studies have reported the mechanisms associated with ncRNAs in PCa. Through the integration of gene chip technology, second-generation sequencing, and data mining, multiple ncRNAs related to the occurrence and progression of PCa have been identified, including microRNA-4429, microRNA-596, microRNA-301a-3p, microRNA-3648, miR-29a-3p, miR-212-5p, miR-30a-5p, miR-324-3p, miR-452-5p, circPHF16, circITCH, lncRNA SNHG7, lncRNA LEF1-AS1, lncRNA HOTAIRM1, and LINC00115, among others [111,115,116,117,118,119,120,121,122,123,124]. It was found that these ncRNAs regulate biological processes, such as cell proliferation, invasion, metastasis, and epigenetics, and play an essential part in PCa development.

Among these ncRNAs, certain microRNAs exert inhibitory effects on the development of PCa through mechanisms such as targeting SLC39A7 and suppressing the Wnt/β-catenin signaling pathway. For example, miR-15a-3p inhibits the proliferation, invasion, and EMT of PCa cells by targeting SLC39A7 and suppressing the Wnt/β-catenin signaling pathway, providing novel therapeutic targets for PCa treatment [125]. Moreover, several studies have revealed that the overexpression of miR-34a exerts inhibitory effects on the Wnt/β-catenin signaling pathway by modulating the transcriptional activity of Wnt1. This, in turn, leads to the suppression of prostate cancer (PCa) cell proliferation, induces cell-cycle arrest at the G2 phase, and ultimately enhances apoptosis in PCa cells [126].

LncRNAs are another important category of ncRNAs in PCa. LncRNA SNHG7 is highly expressed in PCa cells and acts as a sponge for miRNA-324-3p. Through sponge effects, upregulated SNHG7 positively regulates WNT2B, while downregulation of SNHG7 inhibits EMT in PCa, further suppressing cell proliferation, invasion, and metastasis [120]; lncRNA TMPO-AS1 acts as a scaffold to enhance the interaction between CSNK2A1 and DDX3X and activates the Wnt/β-catenin signaling pathway, thereby promoting the bone metastasis of PCa [127]. On the other hand, circPHF16 is downregulated in PCa tissue. It was found that circPHF16 directly interacted with miR-581, which led to the downregulation of no-name finger protein 128 (RNF128), and activated Wnt/β-catenin signals and inhibited metastasis of PCa [119].

In recent studies, there has been a growing interest among researchers in exploring the issue of drug resistance in PCa; there is also research focused on the issue of drug resistance in PCa. Currently, cisplatin is widely used in the treatment of PCa, but cisplatin resistance remains a significant obstacle to cisplatin-based chemotherapy. Studies have found that upregulation of miR-425-5p sensitizes human PCa to cisplatin by targeting GSK3β and inactivating the Wnt/β-catenin signaling pathway, providing a new target for treating PCa, particularly cisplatin-resistant PCa [128].

Immune escape is an important feature in tumor development, which refers to the certain degree of escape ability of tumor cells to attack the immune system, which causes the tumor to be unable to be effectively cleared by the immune system. To suppress PCa progression, anti-tumor immunity is activated, for which cytotoxic T cells are essential. However, lncRNA can induce PD-1 expression, prevent cytotoxic T-cell proliferation and mediate their apoptosis, leading to immune escape from PCa [129]. Therefore, for effective immunotherapy, it is necessary to identify this lncRNA to enhance the potential of immunotherapy. Several studies have shown that in prostate cancer, lncRNA and miRNA can regulate the expression of immune-related genes through multiple mechanisms to influence the immune response and tumor escape. ncRNA may play an important role in regulating the immune escape from prostate cancer, but the specific mechanism needs further investigation.

### 3.5. The Effect of the Interaction between ncRNA and Wnt/β-Catenin Signaling Pathway on Gastric Cancer

Gastric cancer (GC) is a prevalent malignancy, one of the most frequent cancers worldwide, and its incidence and fatality rates are still increasing [130,131,132]. Currently, surgery and chemotherapy are the main treatment methods for GC [132]. However, drug resistance remains a limiting factor in chemotherapy, driving researchers to seek new treatment approaches and targets [133]. Recently, increasing studies have shown that ncRNAs can act as dual-acting regulators to directly or indirectly activate or inhibit the Wnt/β-catenin pathway, involved in GC progression [134].

Research has revealed upregulated expression of certain miRNAs, such as miR-150, miR-876-5p, miR-15a-3p, and miR-216a-3p [135,136], while others, including miR-507, miR-23b-3p, miR-520F-3p, and miR-130a-5p, exhibit downregulated expression [136,137,138]. This may have important implications for the diagnosis and prognosis of GC. The discovery of these miRNAs has important implications in the diagnosis and prognosis of GC. Furthermore, several ncRNAs can interact with specific genes. For instance, miR-100, miR-196b, miR-451, miR-185, miR-130a, miR-122-5p, miR-124, miR-451a, and miR-519b-3p can promote the sensitivity of GC cells to radiotherapy by binding with the corresponding target genes [139].

Additionally, ncRNAs are involved in regulating the Wnt/β-catenin pathway, which has a vital role to play in the development and evolution of GC. For example, circCNIH4 and lncRNA NNT-AS1 inhibited apoptosis in GC cells by inactivating the Wnt/β-catenin pathway, while circSmad4, circHIPK3, circAXIN1, and LINC0035 promoted GC advancement and suggested poor prognosis through activation of the Wnt/β-catenin pathway [140,141,142,143,144,145,146]. Some ncRNAs also regulate GC progression through the ceRNA mechanism. For example, circ0005654 is super-regulated and engaged in the miR-363/sp1/myc/Wnt/β-catenin axis in GC tissues and cells to regulate GC cell proliferation and invasion [147]. Circ0091741 sequesters miR-330-3p, upregulating TRIM14 to stabilize Dvl2, thereby activating the Wnt/β-catenin signaling pathway and promoting autophagy and chemotherapy resistance in GC cells [148]. MiRNA-324-5p activates the Wnt signaling pathway by targeting SUFU, enhancing GC cell proliferation, migration, and inducing EMT [144]. LncRNA VIM-AS1 exhibits high expression in GC tissues and cell lines and promotes GC cell proliferation, migration, invasion, and mesenchymal transition by regulating FDZ1 and activating the Wnt/β-catenin signaling pathway [149]. Additionally, studies have shown that LncRNA SUMO1P3 enhances cell invasion, migration, and cell-cycle processes by strengthening the Wnt/β-catenin signaling pathway [150], whereas miR-6838-5p affects GC cell growth, migration, and invasion through the Wnt/β-catenin signaling pathway by targeting Gprin3 [151].

Although numerous studies have evaluated the underlying molecular mechanisms associated with Wnt/β-catenin-mediated GC and investigated a number of treatments that inhibit this oncogenic pathway, elimination of several drawbacks in therapies targeting Wnt/β-catenin, such as drug resistance or non-responsiveness to signaling requires further investigation. As described in this study, many ncRNAs, as dual-acting regulators, can directly or indirectly activate or inhibit the Wnt/β-catenin pathway, and the interaction between these ncRNA and Wnt/β-catenin signaling pathways seems to be a key mechanism in regulating GC. Thus, targeting the circRNA/lncRNA/miRNA/β-catenin axis may be a promising alternative to other targeted therapies based on Wnt/β-catenin signaling.

## 4. Perspective

Despite significant progress in understanding the mechanisms of action of ncRNAs for Wnt/β-catenin signaling-pathway-associated cancers, many therapeutic challenges remain [152,153]. The regulation of the Wnt/β-catenin signaling pathway by ncRNAs has significant potential value in cancer treatment, but current studies are still in the early stages. Efforts are being made to overcome these challenges by exploring new treatment strategies and methods to open up new avenues for cancer treatment.

The Wnt/β-catenin signaling pathway plays a critical role in various types of cancers, including colorectal cancer, breast cancer, and lung cancer. ncRNAs have been extensively studied and shown to play a key role in regulating cellular biological processes. Here are some suggestions for ncRNA-based therapies targeting the Wnt/β-catenin signaling pathway in cancer:

miRNA-based therapy: Introducing miRNAs that inhibit the activation of the Wnt/β-catenin signaling pathway can effectively suppress the growth and spread of related tumors. For example, miR-34a and miR-200 families have been shown to inhibit β-catenin expression, thereby suppressing the Wnt/β-catenin signaling pathway. Currently, in phase I clinical trials, researchers have successfully delivered synthetic miR-34a mimics to the liver using lipid nanoparticle delivery systems, precisely targeting cancer tissues and regulating the expression of various tumor-suppressor genes such as p53. Therefore, synthesizing miRNA mimics or utilizing delivery systems carrying these miRNAs could be a potential therapeutic strategy.

circRNA regulation: circRNAs have also been found to be associated with the Wnt/β-catenin signaling pathway in cancer. Through techniques such as bioinformatics analysis and high-throughput sequencing, key circRNAs relevant to the Wnt/β-catenin signaling pathway can be identified and validated. Their expression levels and functions in cancer can be experimentally validated. In the functional studies and mechanism elucidation phase, researchers can determine the specific functions and molecular mechanisms of these relevant circRNAs in tumor growth, proliferation, metastasis, and drug resistance. Once the functions and mechanisms of key circRNAs are confirmed, therapeutic strategies targeting circRNAs can be considered. RNA interference (RNAi) and the CRISPR-Cas9 editing system are commonly used techniques for circRNA targeting. RNAi can guide the degradation or inhibition of circRNAs by designing and synthesizing specific small RNA molecules, such as siRNAs or shRNAs. This can be achieved through *in vitro* transfection or *in vivo* delivery of RNAi molecules. The CRISPR-Cas9 editing system can induce DNA double-strand breaks on the circular structure of circRNAs using specific oligonucleotide sequences, leading to their degradation. Additionally, the CRISPR-Cas9 system can be used to genetically edit key regulatory genes to further modulate the activity of the Wnt/β-catenin signaling pathway.

lncRNA-targeted therapy: Many lncRNAs have been found to play important roles in controlling the Wnt/β-catenin signaling pathway. By designing appropriate off-target approaches, the functions of these lncRNAs can be selectively interfered with to regulate the activity of the Wnt/β-catenin signaling pathway. Specifically, targeted interventions using RNAi technology and the CRISPR-Cas9 editing system can selectively reduce or inhibit the expression of relevant lncRNAs. For example, an engineered nanoparticle platform delivering lncAFAP1-AS1 siRNA effectively reversed the radioresistance of TNBC. In addition to nanoparticle-based drug delivery systems, recent research has also proposed the therapeutic potential of using the CRISPR/Cas9 system to edit LncRNA expression in CRC patients, and relevant exploration is underway.

Combination therapy: Combination therapy is a strategy that integrates ncRNA-based therapy with other conventional treatment modalities, including chemotherapy, targeted therapy, and immunotherapy, aiming to enhance the effectiveness of cancer treatment. By simultaneously targeting the Wnt/β-catenin signaling pathway and other oncogenic signaling pathways, combination therapy can comprehensively target cancer and offer several advantages. Firstly, combination therapy can overcome resistance issues. Some tumors develop resistance to chemotherapy drugs, but by utilizing ncRNA-based therapy, tumor sensitivity to drugs can be enhanced, effectively addressing drug resistance. Secondly, ncRNA-based therapy and other conventional treatment modalities typically inhibit tumor growth and spread through different mechanisms. Therefore, combining them can achieve synergistic effects on multiple targets, leading to more effective control of tumor development. Additionally, ncRNA-based therapy generally has lower toxicity and side effects, so combining it with conventional treatment methods can reduce the side effects associated with therapy and improve the quality of life for patients. Finally, combination therapy employs a comprehensive treatment approach, combining different treatment modalities that can act on tumors at different levels and time points. This integrated treatment strategy can comprehensively suppress tumors and improve the overall effectiveness of treatment.

The diverse mechanisms by which ncRNAs regulate the Wnt/β-catenin signaling pathway represent potential therapeutic targets for cancer treatment. Further research is needed to identify specific ncRNAs and their potential mechanisms in different types of cancer, as well as to develop more effective delivery strategies. Overall, studying ncRNAs in Wnt/β-catenin signaling-pathway-associated cancers has great potential to improve cancer diagnosis and treatment.

## Figures and Tables

**Table 1:** Research overview of ncRNAs and Wnt/β-catenin signaling pathway in cancers (Activation: activation of the Wnt signaling pathway; Suppression: inhibition of the Wnt signaling pathway).

Cancer Type	ncRNAs	Expression	Mechanisms	Functions to Wnt Pathway	PMID
Breast cancer	miR-125b	Up	Wnt/β-catenin	Activation	30950326
	miR-296-3p	Down	SOX4	Suppression	34785625
	miR-96-5p	Down	CTNND1	Suppression	31913290
	miR-9501	Up	β-catenin	Suppression	32373971
	miR-548c-5p	Down	Wnt1	Suppression	32329856
	miR-516a-3p	Down	Pygopus2	Suppression	31273950
	miR-454-3p	Up	RPRD1A	Activation	30809286
	miR-449b-5p	Down	CREPT	Suppression	30738779
	miR-429	Down	Wnt/β-catenin	Suppression	32961031
	miR-34a	Up	Wnt3/Wnt1	Activation	30779084
	miR-340-5p	Down	LGR5	Suppression	30300682
	miR-296	Down	FGFR1	Suppression	31841196
	miR-27a	Up	GSK-3β	Activation	33025840
	miR-216a	Down	Wnt/β-catenin	Suppression	30864744
	miR-193b	Down	c-Met	Suppression	34863149
	miR-190	Down	SOX9	Activation	30658681
	miR-135	Down	Wnt/β-catenin	Suppression	35730603
	miR-130a-3p	Down	NRARP	Suppression	35797350
	miR-124-3p.1	Up	Axin1	Activation	32125723
	lncRNA MICAL2-1	Down	miR-25/DKK3	Suppression	34970696
	lncRNA RP11-283G6.5	Down	miR-188-3p/TMED3	Suppression	34416888
	lncRNA RUSC1-AS-N	Up	Wnt1/β-catenin	Activation	30569097
	lncRNA RBM5-AS1	Up	Wnt/β-catenin	Activation	35110544
	lncRNA HOTTIP	Up	miR-148a-3p/WNT1	Activation	32307830
	lncRNA HOTTIP	Up	Wnt/β-catenin	Activation	30676763
	lncRNA H19	Up	miR-340-3p/YWHAZ	Activation	30676763
	lncRNA C5orf66-AS1	Up	miR-149-5p/CTCF/CTNNB1	Activation	35499320
	lncRNA ASMTL-AS1	Down	miR-1228-3p/SOX17	Suppression	34006305
	lncRNA LUCAT1	Up	miR-5582-3p/TCF7L2	Activation	31300015
	lncRNA CCAT1	Up	miR-204/211/miR-148a/152/ANXA2	Activation	31695775
	LINC01287	Up	Wnt/β-catenin	Activation	31173295
	LINC01234	Up	miR-525-5p/MEIS2	Activation	34173712
	circ_0008784	Up	miR-506-3p/CTNNB1	Activation	36436315
	circ-ITCH	Down	miR-214/miR-17	Activation	30509108
	circARL8B	Up	miR-653-5p/HMGA2	Activation	34050452
	circABCC4	Up	miR-154-5p	Suppression	34050452
Lung cancer	miR-590	Down	YAP1	Suppression	35031966
	miR-448	Down	SATB1	Activation	32525527
	miR-489-3p	Up	USP48	Activation	35413838
	miR-421	Up	HOPX	Activation	31115507
	miR-23B	Down	RUNX2	Suppression	32495614
	miR-20b	Down	APC	Activation	31894264
	miR-1b-19p	Down	MYPT3	Suppression	33964297
	miR-147b	Down	RPS15A	Suppression	31665807
	miR-103	Up	KLF7	Activation	32582959
	miR-100	Down	HOXA1	Suppression	32364673
	miR-520a	Up	RRM2	Suppression	33859925
	lncRNA SNHG11	Up	miR-4436a/CTNNB1	Activation	32239719
	lncRNA FLVCR1-AS1	Up	Wnt/β-catenin	Suppression	30697812
	lncRNA-SNHG7	Down	miR-181/cbx7	Suppression	32201260
	lncRNASEH1-AS1	Up	miR-516a-5p/FOXK1	Activation	35166053
	lncRNA SNHG20	Up	miR-197/TCF/LEF1	Activation	31957836
	lncRNA PVT1	Up	miR-361-3p/SOX9	Activation	32197208
	lncRNA JPX	Up	miR-33a-5p/Twist1	Activation	32197208
	lncRNA HJURP	Up	β-catenin	Suppression	31115012
	LncRNA DSCAM-AS1	Up	miR-577/HMGB1	Activation	32386483
	lncRNA AWPPH	Up	Wnt/β-catenin	Activation	32386483
	LncDBH-AS1	Down	miR-155/AXIN1	Activation	33506901
	LINC01006	Up	miR-129-2-3p/CTNNB1	Activation	33753463
	LINC00942	Up	miR-5006-5p/FZD1	Activation	34253104
	LINC00669	Up	Wnt/β-catenin	Activation	36621836
	LINC00326	Up	miR-657/DKK2	Suppression	36747258
	LINC00673-v4	Up	DDX3/CK1ε	Activation	31235588
	circ-EIF3I	Up	miR-1253/NOVA2	Activation	36193788
	has_circ_0017109	Up	miR-671-5p/FZD4	Activation	36434577
	has_circ_0001946	Up	miR-135a-5p/SIRT1	Activation	30841451
	hsa_circ_0066903	Down	miR-3681-3p/miR-3909/GSK3B	Suppression	35821283
	hsa_circ_0007059	Down	miR-378	Suppression	31351967
	has_circ_0006427	Down	miR-6783-3p/DKK1	Suppression	30470570
	circ-ZNF124	Up	miR-498/YES1	Suppression	33186139
	circVAPA	Up	miR-876-5p/WNT5a	Activation	33619796
	circ-PGC	Up	miR-2-532p/FOXR3	Activation	34494941
	circ_0067934	Up	miR-1182/KLF8	Activation	32768951
Colorectal cancer	miR-621	Down	LEF1	Suppression	36087740
	miR-576-5p	Up	Wnt5a	Activation	33300054
	miR-532-3p	Down	ETS3/TGM1	Suppression	31570702
	miR-501-3p	Up	APC	Activation	31364752
	miR-381	Down	SPIN1	Activation	34753384
	miR-377-3p	Down	ZEB2/XIAP	Suppression	32220639
	miR-30-5p	Down	USP2	Suppression	30338942
	miR-19a-3p	Up	FOXF2	Suppression	32103872
	miR-188	Up	FOXL1	Activation	37305399
	miR-183-5p	Up	RCN2	Suppression	30896885
	miR-144-3p	Down	BCL6	Suppression	32206063
	miR-103/107	Up	Axin2	Activation	31273221
	miR-6125	Down	YTHDF2	Activation	34709763
	miR-520e	Down	AEG-1	Suppression	31574178
	LINC00665	Up	miR-214-3p/CTNNB1	Activation	33865827
	lncRNA TUG1	Up	miR-542-3p/TRIB2	Activation	34030715
	lncRNA PART1	Up	miR-150-5p/miR-520h/CTNNB1	Activation	31669140
	lncRNA NEAT1	Up	miR-486-5p/NR4A1	Activation	33337350
	lncRNA NEAT1	Up	miR-34a/SIRT1	Activation	30312725
	lncRNA HCG18	Up	miR-1271/MTDH	Activation	31854468
	lncRNA ADAMTS9-AS1	Down	Wnt/β-catenin	Suppression	32889785
	LINC01315	Up	Wnt/β-catenin	Activation	35322763
	LINC00963-v2/-v3	Down	miR-143/miR-217/miR-512/APC/Axin	Suppression	36804476
	LINC00365	Up	CDK1	Activation	31544991
	circ_0082182	Up	miR-411/miR-1205	Activation	33596920
	hsa_circ_0026628	Up	miR-346/SP1	Activation	34420031
	hsa_circ_0068464	Up	miR-383	Activation	35168468
	hsa_circ_0009361	Down	miR-582/APC2	Suppression	31109967
	hsa_circ_0005615	Up	miR-149-5p/TNKS	Activation	32393760
	hsa_circ_0005075	Up	Wnt/β-catenin	Activation	31081084
	circRASSF2	Up	miR-195-5p/FZD4	Activation	33929991
	circPTK2	Up	miR-136-5p/YTHDF1	Activation	34974791
	circ-IGF1R	Up	miR-362-5p/HMGB3	Activation	36542208
	circIFT80	Up	miR-142/miR-568/miR-634/CTNNB1	Activation	35783013
	circAGFG1	Up	miR-4262/miR-185-5/pYY1/CTNNB1	Activation	32681092
	circ-ACAP2	Up	miR-143-3p/FZD4	Activation	34085707
	circ_0026344	Down	miR-183	Suppression	31608699
Prostate cancer	miR-4429	Down	DLX1	Suppression	33740948
	miR-596	Down	β-catenin	Suppression	33387246
	miR-15a-3p	Down	SLC39A7	Suppression	31135177
	miR-34a	Down	Wnt1	Suppression	32894541
	miR-425-5p	Down	GSK3β	Suppression	31502580
	miR-653-5p	Down	SOX30	Suppression	31889959
	miR-95-3p	Up	DKK3	Activation	30779066
	lncRNA SOX2-OT	Up	miR-452-5p/HMGB3	Suppression	32407168
	lncRNA SNHG12	Up	miR-195	Activation	30945357
	lncRNA HOTTIP	Up	Wnt/β-catenin	Suppression	30809864
	LINC00115	Up	miR-212-5p/FZD5	Activation	34697900
	circPHF16	Down	miR-581/RNF128	Suppression	36503162
Gastric cancer	miRNA-150	Up	SUFU	Activation	33848981
	miR-520f-3p	Down	SOX9	Suppression	32277152
	miR-507	Down	CBX4	Suppression	35819589
	miR-324-5p	Up	SUFU	Activation	33017570
	miR-6838-5p	Down	GPRIN3	Suppression	33254176
	miR-192/-215	Up	APC	Activation	32091625
	miR-188-5p	Up	PTEN	Activation	31138169
	miR-195-5p	Down	YAP	Activation	31378888
	miR-381/miR-489	Down	CUL4B	Suppression	30483755
	miR-675	Up	PITX1	Activation	31260797
	LINC00355	Up	Wnt/β-catenin	Activation	32894544
	lncRNA NNT-AS1	Up	miR-142-5p/SOX4	Activation	32468065
	lncRNA VIM-AS1	Up	miR-8052/FDZ1	Activation	33173977
	lncRNA SUMO1P3	Up	Wnt/β-catenin	Activation	33179980
	LINC01225	Up	Wnt/β-catenin	Activation	31460694
	LINC01503	Up	Wnt/β-catenin	Activation	32207034
	lncRNA H19	Up	β-catenin	Activation	34348271
	lncRNA MIR4435-2HG	Up	DSP	Activation	31484163
	lncRNA NCK1-AS1	Up	miR-22-3p/BCL9	Activation	33974352
	lncRNA SNHG11	Up	miR-483-3p/miR-1276/CTNNB1/ATG12	Activation	33068778
	lncRNA ZEB2-AS1	Up	Wnt/β-catenin	Activation	30635820
	lncRNA ZFAS1	Up	miR-200b/Wnt1	Activation	30999814
	LOC100505817	Down	Wnt/β-catenin	Activation	34385891
	LOC285194	Down	Wnt/β-catenin	Suppression	31991056
	circ0005654	Up	miR-363/sp1	Activation	34499009
	circ_0091741	Up	miR-330-3p/TRIM14	Activation	36323918
	circ-SFMBT2	Up	miR-885-3p/CHD7	Activation	34387601
	cir-ITCH	Down	miR-17	Suppression	33060778
	hsa_circ_0001649	Down	miR-20a/ERK	Suppression	32212290

## Data Availability

Not applicable.
